# MyoAAV-delivered sup-tRNA increases full-length dystrophin expression

**DOI:** 10.1016/j.gendis.2025.101666

**Published:** 2025-05-03

**Authors:** Xiuyi Ai, Yue Chang, Ruo Wu, Jie Liu, Pei Zhang, Yayu Wang, Zhuoyin Zheng, Shu Zhang, Yongchang Chen, Shiwen Wu

**Affiliations:** a Department of Neurology, First Medical Center of Chinese PLA General Hospital, Beijing 100853, China; b State Key Laboratory of Primate Biomedical Research, Institute of Primate Translational Medicine, Kunming University of Science and Technology, Kunming, Yunnan 650500, China; c Yunnan Key Laboratory of Primate Biomedical Research, Kunming, Yunnan 650500, China; d Faculty of Life Science and Technology, Kunming University of Science and Technology, Kunming, Yunnan 650500, China

Duchenne muscular dystrophy (DMD) is a fatal X-chromosome-linked genetic disease caused by dystrophin gene mutations, including nonsense mutations.^1^ Nonsense mutations are caused by the introduction of premature termination codons, which prevent translation of full-length proteins. Read-through therapies show potential for addressing DMD's genetic basis; however, issues such as non-specific amino acid insertions, gene-editing delivery challenges, and clinical safety concerns have limited their progress.^2^^,^^3^ To address nonsense mutations, nonsense suppressor transfer RNAs (sup-tRNAs) have been proposed as a genetic therapy approach.^3^, ^4^, ^5^ In this study, we propose a new MyoAAV-delivered suppressor tRNA (sup-tRNA) strategy to restore dystrophin expression. Our approach specifically targets nonsense mutations in mdx mice and patient-derived myoblasts and cardiomyocytes, significantly increasing dystrophin levels, especially in the heart (up to 61.43% when combined with CC-90009). This combination alleviates dystrophic symptoms and improves read-through efficiency, likely by reducing translation termination factor activity. These findings highlight the potential of sup-tRNA in DMD and other nonsense mutation-related diseases.

Previous studies have shown that sup-tRNAs rescue nonsense mutations and increase protein function under endogenous regulation (Fig. 1A). We selected a set of near-cognate natural tRNAs carrying six different amino acids and mutated their anticodons to 5′-AUU-3′, which were completely base-pairing with the stop codon UAA (Fig. 1B and Table S1). To evaluate the read-through efficiency of the various sup-tRNAs, we designed a plasmid containing the full-length mCherry sequence with a UAA mutation at the codon 67 site (Fig. 1C). Western blotting and fluorescence microscopy showed that in co-transfected HEK293 cells, all sup-tRNAs successfully increased full-length mCherry, with sup-tRNA^Y^ exhibiting higher efficiency compared to the other sup-tRNAs (Fig. 1D, S1). These suggest that sup-tRNA can accurately recognize and induce readthrough of the PTC in the mutant mRNA. We selected sup-tRNA^Y^ with higher efficiency for further research.

**Figure 1:**
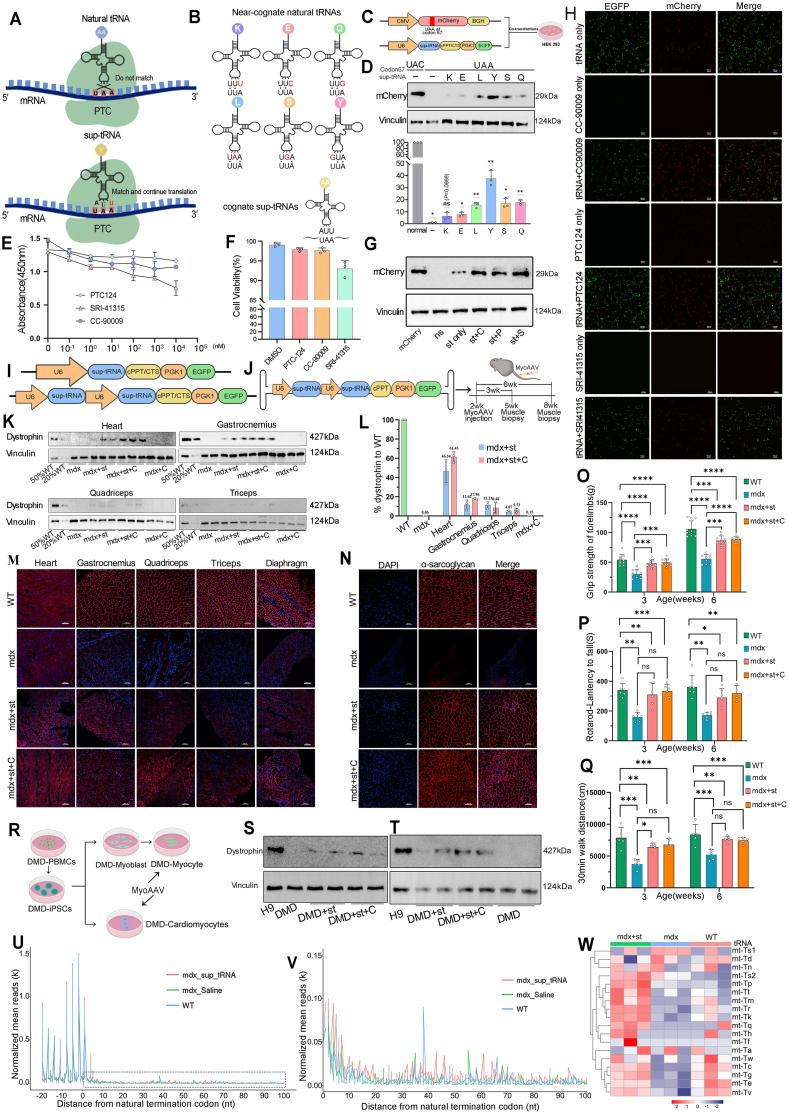
MyoAAV-delivered sup-tRNA increases full-length dystrophin expression.

To improve therapeutic efficiency, we conducted a test to identify small-molecule compounds capable of improving the read-through efficiency of sup-tRNAs. Three selected small-molecule drugs, namely PTC124, CC-90009, and SRI-41315, were chosen for this purpose (Fig. S2). First, we examined the impact of these compounds on cell viability. Treatment with increasing concentrations of SRI-41315 (ranging from 0.1 nM to 104 nM) for 48 h resulted in reduced cell viability. Notably, SRI-41315 exhibited higher toxicity to HEK293 cells than PTC124 and CC-90009 (Fig. 1E, F). Additionally, we also observed that with exposure to high concentrations of SRI-41315 led to slowed cell growth and detachment of some cells from the culture dishes. Furthermore, to explore the combined effect of small-molecule compounds and sup-tRNA therapy, transfected HEK293 cells were exposed to DMSO or 0.1 μM CC-90009 or 5 μM PTC124 or 5 μM SRI-41315 for 72 h. Western blotting confirmed an increase in mCherry expression with the addition of each of the three small molecules (Fig. 1G). Fluorescence microscope images further suggests that the enhanced stop codon read-through sup-tRNA + molecule is caused by the synergy between the 2 agents as the read-through efficiency of small molecule compounds alone is very low (Fig. 1H). Taken together, these findings suggest that all three small-molecule drugs can enhance the sup-tRNA read-through efficiency, leading to the restoration of full-length mCherry. Consequently, we hypothesized that reduced levels of translation termination factors and the inhibition of the release factor prolong translational pauses at PTCs, enabling sup-tRNA-mediated amino acid insertion and read-through.

To enhance sup-tRNA expression and read-through efficiency, we generated a construct containing two copies of the sup-tRNA^Y^ expression cassette, based on previously reported studies demonstrating that a multimeric structure can improve sup-tRNA expression in vivo (Fig. 1I and Table S2). The 2 × sup-tRNA construct was then packaged into a muscle-targeted recombinant AAV (MyoAAV2A) vector to facilitate efficient delivery to affected muscle tissue and promote read-through of premature stop codons. For treatment, we selected 2-week-old mdx mice and systemically administered MyoAAV-sup-tRNA at a dose of 10 × 10^13^ vg/kg via intravenous injection for either 3 or 6 weeks (Fig. 1J). Simultaneously, CC-90009 was intraperitoneally injected into mice at a dose of 2.5 mg/kg. Post-muscle biopsy, we observed that EGFP was predominantly distributed in muscle tissues and kidneys, with slight distribution in the lungs, brain, and liver (Fig. S3), which is consistent with previous reports. Muscle biopsy samples were then used to estimate read-through efficiency by western blotting. It revealed the widespread expression of full-length dystrophin protein in different muscles, with the highest levels in the heart. In the sup-tRNA^Y^ group, full-length dystrophin expression was 46.54%, 11.62%, 11.21%, and 4.87% in the heart, gastrocnemius, quadriceps, and triceps, respectively. In the combination group of CC-90009 and sup-tRNA^Y^, full-length dystrophin expression reached 61.43%, 17.96%, 8.48%, and 5.72% in the heart, gastrocnemius, quadriceps, and triceps, respectively. Statistical analysis using one-way ANOVA followed by Tukey's multiple comparisons test revealed that dystrophin expression in the combination group was significantly higher than in the sup-tRNA^Y^ alone group across all muscle types (adjusted *p* values shown in Supplementary Table S3). In contrast, no significant dystrophin expression was observed in the CC-90009 group alone (Fig. 1K, L). Notably, immunofluorescence staining confirmed the clear restoration of dystrophin expression (Fig. 1M). Additionally, hematoxylin and eosin (H&E) staining indicated that the muscle morphology was partially corrected, and the infiltration of inflammatory cells was reduced (Fig. S4A). Sirius Red staining qualitatively showed a reduction in the proportion of collagen fibers in the treated groups compared to controls (Fig. S4B). Immunofluorescence staining also showed that sup-tRNA could recover the expression of α-sarcoglycan, one of the dystrophin-related proteins, to maintain muscle cell membrane stability (Fig. 1N). In particular, sup-tRNA-treated mice exhibited noticeable improvements in overall behavioral phenotypes. Specifically, there were no deaths, and body weights remained stable throughout the treatment period (Fig. S5). Moreover, quantitative assessments revealed significant enhancements in grip strength, treadmill endurance, and spontaneous activity (Fig. 1O, P, Q), supporting the observed functional rescue. We employed a nonsense mutation DMD induced pluripotent stem cells (iPSC) line (DMD: NM_004006: c.790G > T p. G264∗) for differentiation, following a well-established differentiation process from stem cells to mature myotubes or cardiomyocytes, as previously reported (Fig. 1R, S6). Immunofluorescence staining confirmed the expression of dystrophin in the DMD patient's differentiated myoblasts treated with sup-tRNA^Y^ or sup-tRNA^Y^ combined with CC90009 (Fig. S7A). Western blotting experiments validated the restoration of dystrophin levels, both with and without CC-90009 treatment (Fig. 1S). Similar outcomes were observed in DMD patient's iPSC differentiated cardiomyocytes (Fig. 1T, S7B). Taken together, our findings demonstrate that sup-tRNAs are effective in inducing endogenous read-throughs in human-derived myotubes and cardiomyocytes.

Importantly, we did not observe any adverse reactions or other relevant toxicities after the administration of MyoAAV-sup-tRNA^Y^. As mentioned earlier, mice in the different groups exhibited normal stools, weight gain, shiny hair, diet, normal activity, and mental state, with no recorded deaths during the experiment (Fig. S5). In order to gain insights into the global read-through induced by sup-tRNA^Y^ and assess the safety profile of this approach, we conducted ribosome profiling (Ribo-seq) on gastrocnemius muscles from wild-type (WT), Myo AAV-sup-tRNA^Y^ treated mice and untreated (with saline) mdx mice (*n* = 3 each). Our findings revealed that ribosome-protected mRNA fragments (RPFs) quantified using biological replicates were well correlated (Fig. 1U, V). Differential expression analysis revealed significant alterations in mitochondrial tRNA profiles among the three groups. Specifically, several mitochondrial tRNAs showed normalized or distinct expression patterns in the Myo AAV-sup-tRNA^Y^ treated mdx mice compared to untreated mdx controls (Fig. 1W). These findings suggest that sup-tRNA therapy may modulate not only PTC readthrough but also overall mitochondrial translational regulation, contributing to improved muscle function. Gene Ontology (GO) analysis further demonstrated that in the muscles of mice treated with sup-tRNA, highly enriched transcripts were associated with processes such as base pairing, responses to amino acids, mitochondrial metabolism, and extracellular matrix composition (Fig. S8).

## CRediT authorship contribution statement

**Xiuyi Ai:** Writing – original draft, Methodology, Formal analysis, Data curation, Conceptualization. **Yue Chang:** Formal analysis, Data curation. **Ruo Wu:** Methodology, Data curation. **Jie Liu:** Methodology. **Pei Zhang:** Writing – review & editing. **Yayu Wang:** Data curation. **Zhuoyin Zheng:** Data curation. **Shu Zhang:** Writing – review & editing, Conceptualization. **Yongchang Chen:** Writing – review & editing, Funding acquisition, Conceptualization. **Shiwen Wu:** Writing – review & editing, Funding acquisition, Conceptualization.

## Ethics declaration

This study was approved by the Ethics Committee of the Chinese PLA General Hospital (approval number KY2121-001).

## Funding

This work was supported by the National Key Research and Development Program of China (No. 2022YFC2703600) and the National Natural Science Foundation of China (No. 81930121).

## Conflict of interests

The authors declared no conflict of interests.
