# Supplementary material for: MyoAAV-delivered sup-tRNA increases full-length dystrophin expression
Source: Genes Dis. 2025 May 3;12(5):101666. doi: 10.1016/j.gendis.2025.101666 (PMC12148385; doi:10.1016/j.gendis.2025.101666)
Supplement: Multimedia component 1 [file mmc1.pdf]

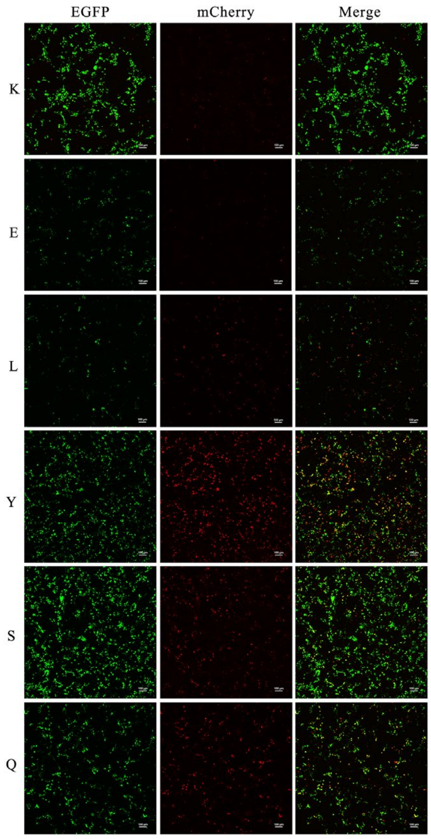

Figure S1. sup-tRNAs effectively mediate read-through of UAA nonsense mutations in vitro.
Fluorescence microscope images of mCherry in HEK293 after 48 h of transfection (EGFP in green, mCherry in red). Scale bars, 100 μm.

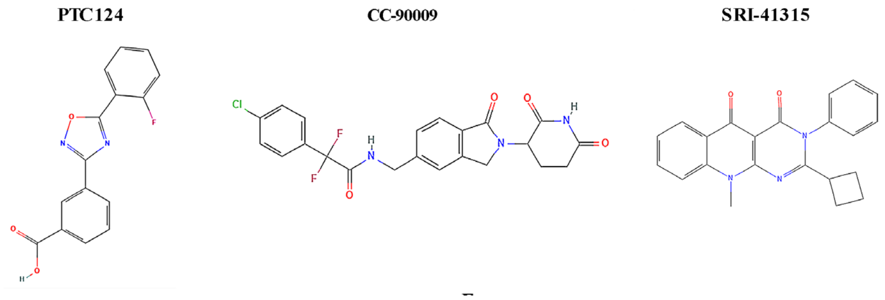

Figure S2. Structures of Small molecule compounds.

Structures of PTC124, CC-90009, and SRI-41315.

PTC124, identified as an apparent competitive inhibitor of productive release factor complex (RFC) binding, inhibits release factor activity and facilitates the recognition of near-cognate tRNAs at PTCs.[1-3]

CC-90009, a novel cereblon E3 ligase modulator, induces the proteasomal degradation of eRF3a and eRF3b, thereby reducing eRF1 levels and upregulating UPF1 and NMD suppression.[4;5]

SRI-41315 binds eRF3 to the PTC in the A site of the ribosome thereby depleting eRF3 and reducing the abundance of the termination factor eRF1 and potentiates aminoglycoside-mediated read-through.[6;7]

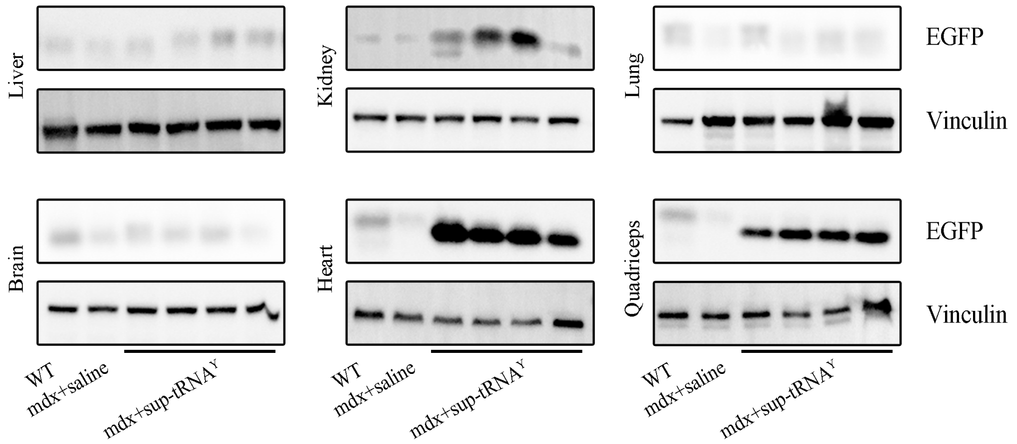

Figure S3. Myo AAV-delivered sup-tRNA is more highly expressed in muscles than other tissues.

Western blot analysis showed EGFP expression in the heart, quadriceps and kidney, but less in the liver, lung and brain, of mdx mice, six weeks after systemic injection of sup-tRNA packaged in Myo AAV.

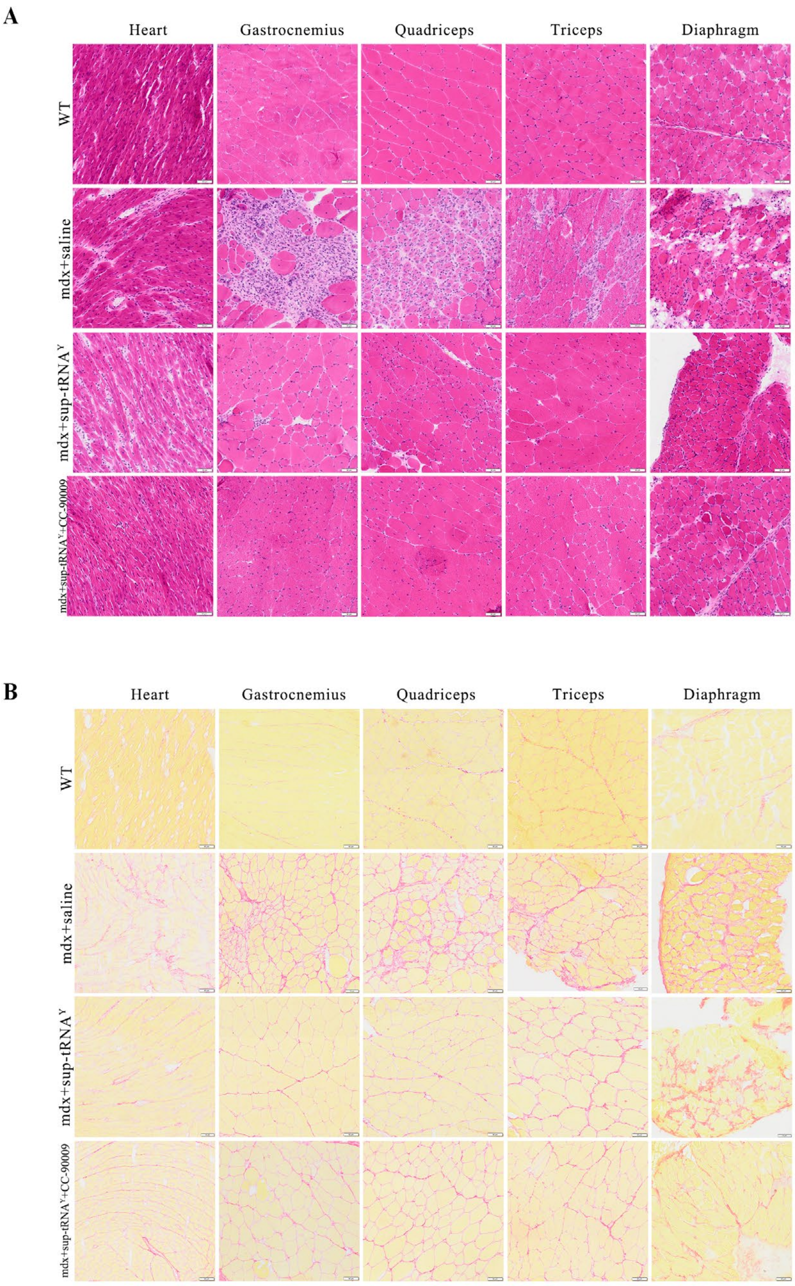

Figure S4. Sup-tRNA with or without CC-90009 both improve the pathological phenotype in injected mdx mice.

(A) Hematoxylin-eosin(HE) staining of heart, gastrocnemius, quadriceps, triceps and diaphragm in WT, mdx+saline, mdx+sup-tRNA^{Y}, mdx+sup-tRNA^{Y}+CC-90009 mice. Scale bar, 50 µm. (B) Sirius red staining of heart, gastrocnemius, quadriceps, triceps and diaphragm in four groups mice. Scale bar, 50 µm.mice.

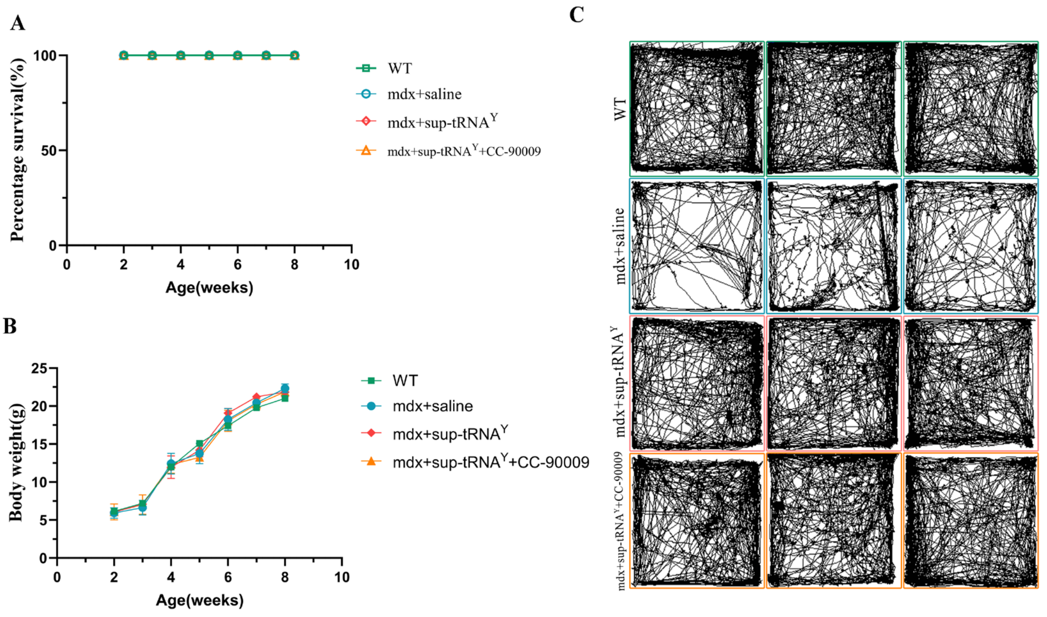

Figure S5.

Sup-tRNA with or without CC-90009 both improve the motor function of injected mdx mice.

(A) Survival rates of different mice groups. (B) Body weight curves of different mice groups. (C) The analysis of movement trajectory within 30 minutes recording of four groups' mice. Data are mean ± SD. n = 6 for each group.

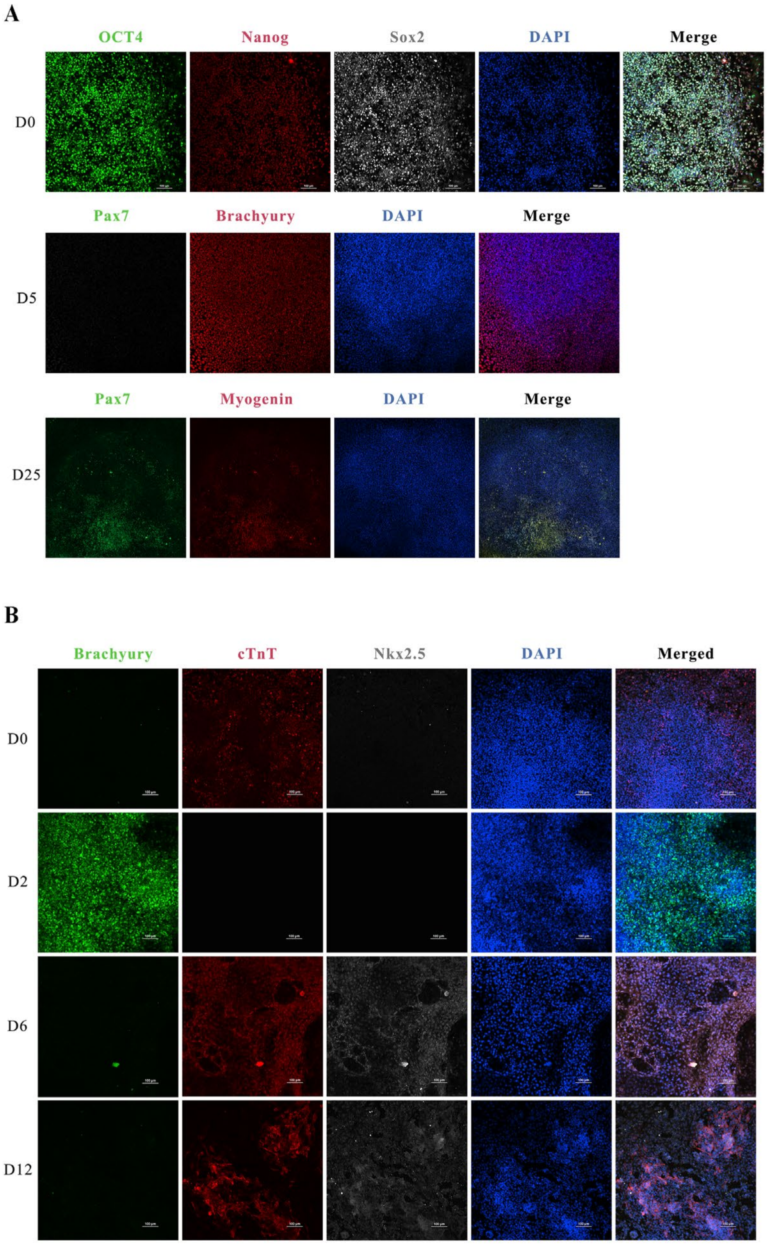

Figure S6. Differentiation of myotubes and cardiomyocytes in vitro.

(A) Representative phase-contrast and immunofluorescence staining images of differentiating DMD iPSC-derived myotubes. Day 0: pluripotency markers OCT4, Nanog, and Sox2; Day 5: Pax7 and Brachyury; Day 25: Pax7 and Myogenin.

(B) Immunofluorescence staining of Brachyury, cTnT, and Nkx2.5 in DMD iPSC-derived cardiomyocytes at different stages (Day 0, Day 2, Day 6, and Day 12).

Differentiation protocols were based on established methods previously described in references.[8-10] Immunostaining was performed according to standard procedures. Scale bar: 100 μm.

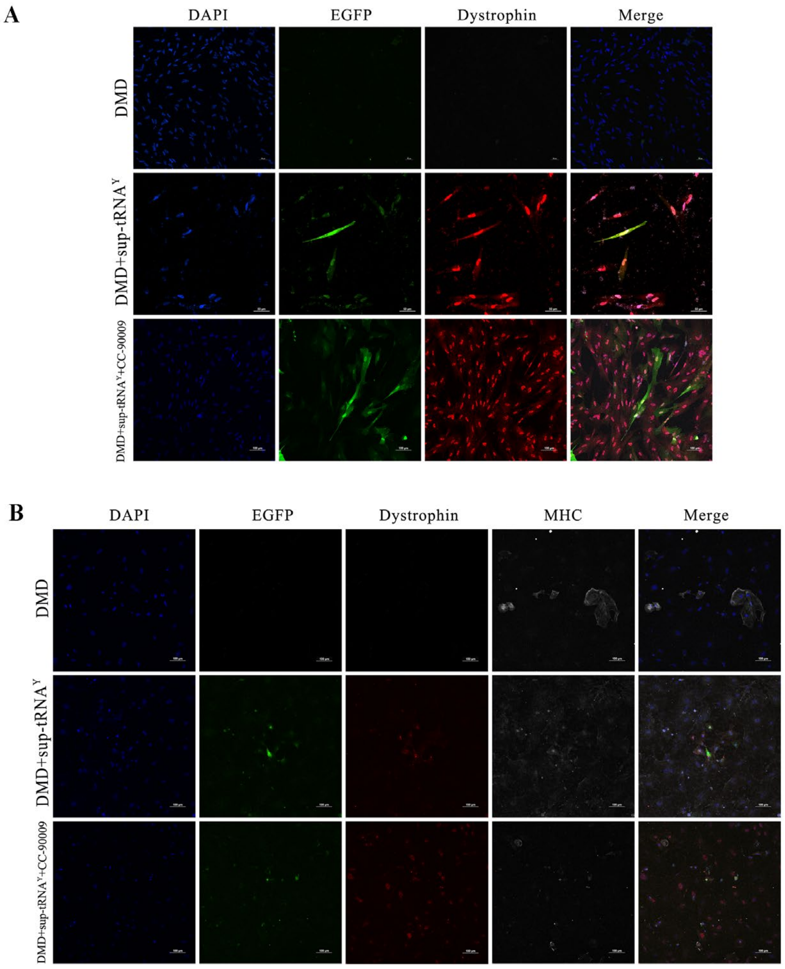

Figure S7. Sup-tRNA increased dystrophin expression in a DMD patient's differentiated myotubes and cardiomyocytes.

(A) Fluorescence staining of dystrophin in different groups' myotubes. (B) Fluorescence staining of dystrophin in different groups' cardiomyocytes. Immunostaining was performed using the protocol described in previous reports.[11;12]

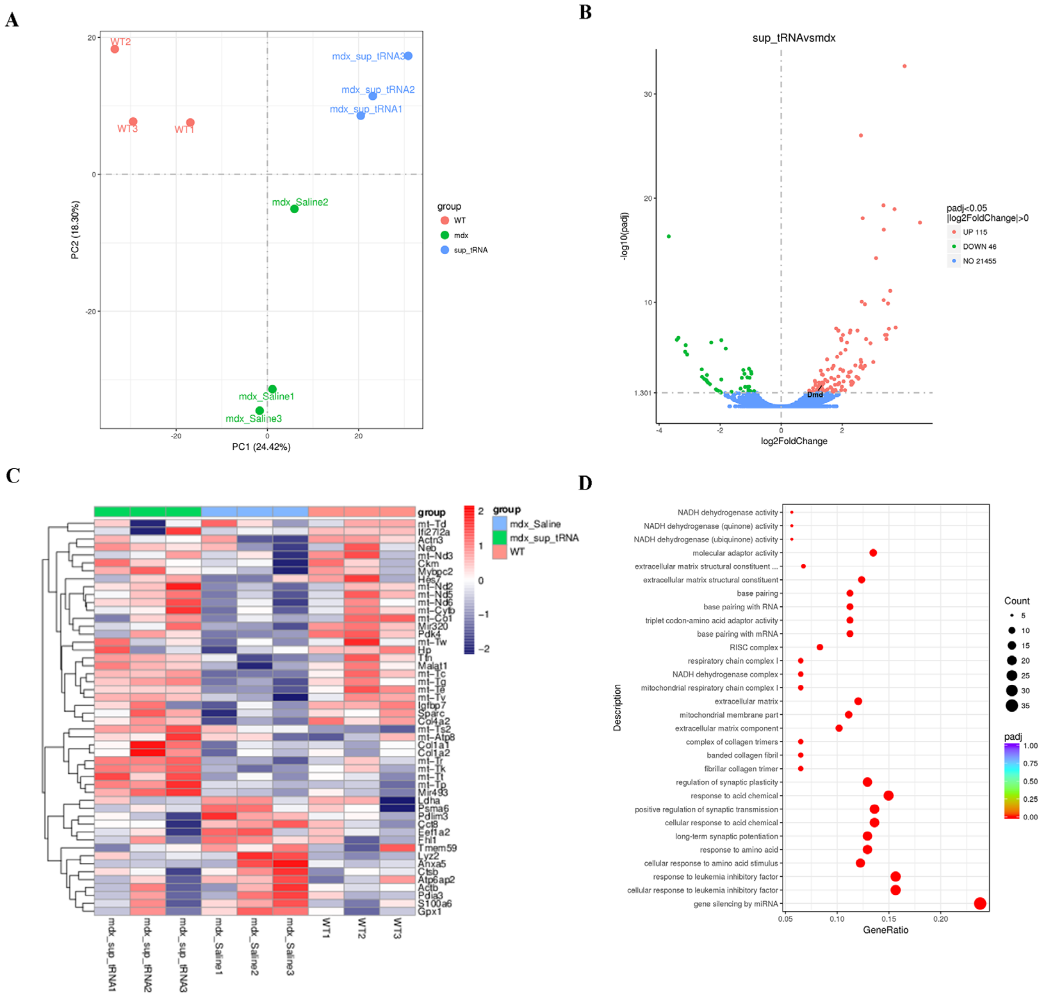

Figure S8. Ribosome profiling explored the effect on global readthrough.

(A) Principal component analysis (PCA) plot showing variation among 12 378 samples in three groups (WT, treated and untreated mdx mice; n=3). (B) Volcanic map showing dmd gene in sup-tRNA^{Y} treated mdx mice; padj<0.005. (C) Heatmap showing the top 50 genes with the largest difference in expression ratio in three groups. (D) Gene ontology (GO) terms enrichment of differentially expressed showed that sup-tRNA affected base pairing, response to amino acid, mitochondrial metabolism and extracellular matrix composition.

Table S1. Sup-tRNA gene sequences.

sup-tRNA name	sup-tRNA gene sequence (5' to 3')
sup-tRNA^{K}	GCCCGGATAGCTCAGTCGGTAGAGCATCAGACTTTAAATCTGAGGGTCCAGGGTTCAAGTCCCTGTTCGGGCG
sup-tRNA^{E}	TCCCTGGTGGTCTAGTGGCTAGGATTCGGCGCTTTAACCGCCGCGGCCCGGGTTCGATTCCCGGCCAGGGAA
sup-tRNA^{Q}	GGTCCCATGGTGTAATGGTTAGCACTCTGGACTTTAAATCCAGCAATCCGAGTTCGAATCTCGGTGGGACCT
sup-tRNA^{L}	ACCAGGATGGCCGAGTGGTTAAGGCGTTGGACTTTAGATCCAATGGACATATGTCCGCGTGGGTTCGAACCCCACTCCTGGTA
sup-tRNA^{S}	GTAGTCGTGGCCGAGTGGTTAAGGCGATGGACTTTAAATCCATTGGGGTTTCCCCGCGCAGGTTCGAATCCTGCCGACTACG
sup-tRNA^{Y}	CCTTCGATAGCTCAGTTGGTAGAGCGGAGGACTTTAGATCCTTAGGTCGCTGGTTCGAATCCGGCTCGAAGGA

The design process of Sup-tRNA gene sequences was carried out in accordance with the reported articles.[13;14] Natural tRNA sequences were obtained from GtRNAdb (http://gtrnadb.ucsc.edu/), and antisense codons were mutated to UAA. Evaluation of the sup-tRNAs was conducted using tRNAscan-SE (http://trna.ucsc.edu/tRNAscan-SE/).

Table S2. Nucleotide sequences of 1× or 2× copies of the sup-tRNA^{Y}.

1× sup-tRNA^{Y}	gagggcctatttcccatgattccttcatatttgcatatacgatacaaggctgttagagagataattggaattaatttgactgtaaacacaaagatattagtacaaaatacgtga<br>cgtagaaagtaataatttcttgggtagtttgcagttttaaaattatgttttaaaatggactatcatatgcttaccgtaacttgaaagtatttcgatttcttggctttatatatcttgtgg<br>aaaggacgaaacaccgCCTTCGATAGCTCAGTTGGTAGAGCGGAGGACTTTAGATCCTTAGGTCGCTGGTTCG<br>AATCCGGCTCGAAGGAtttttttaaagaattctcgacctcgagacaaatggcagtattcatccacaattttaaaagaaaaggggggattggggggtacagt<br>gcaggggaaagaatagtagacataatagcaacagacatacaaactaaagaattacaaaaacaaattacaaaaattcaaaattttcgggtttattacagggacagcagag<br>atccactttggccgcggctcgagggggttggggttgcgccttttccaaggcagccctgggtttgcgcagggacgcggctgctctgggcgtggttccgggaaacgcag<br>cggcgccgaccctgggactcgcacattcttcacgtccgttcgcagcgtcacccggatcttcgccgctacccttgtgggccccccggcgacgcttcctgctccgcccta<br>agtcgggaaggttccttgcggttcgcggcgtgccggacgtgacaaacggaagccgcacgtctcactagtaccctcgcagacggacagcgccagggagcaatggca<br>gcgcgccgaccgcgatgggctgtggccaatagcggctgctcagcagggcgcgccgagagcagcggccgggaaggggcggtgcgggaggcggggtgtggggc<br>ggtagtgtgggccctgttcctgcccgcgcggtgttccgcattctgcaagcctccggagcgcacgtcggcagtcggctccctcgttgaccgaatcaccgacctctctccc<br>caggggggatccatggtgagcaagggcgaggagctgttcaccggggtggtgcccatcctggtcgagctggacggcgacgtaaacggccacaagttcagcgtgtccg<br>gcgagggcgagggcgatgccacctacggcaagctgaccctgaagttcatctgcaccaccggcaagctgcccgtgccctggcccaccctcgtgaccaccctgaccta<br>cggcgtgcagtgcttcagccgctaccccgaccacatgaagcagcacgacttcttcaagtccgccatgcccgaaggctacgtccaggagcgcaccatcttcttcaagga<br>cgacggcaactacaagacccgcgccgaggtgaagttcgagggcgacaccctggtgaaccgcatcgagctgaagggcatcgacttcaaggaggacggcaacatcct<br>ggggcacaagctggagtacaactacaacagccacaacgtctatatcatggccgacaagcagaagaacggcatcaaggtgaacttcaagatccgccacaacatcgag<br>gacggcagcgtgcagctcgccgaccactaccagcagaacacccccatcggcgacggccccgtgctgctgcccgacaaccactacctgagcacccagtccgccctg<br>agcaaagaccccaacgagaagcgcgatcacatggtcctgctggagttcgtgaccgccgccgggatcactctcggcatggacgagctgtacaagtaaa
2× sup-tRNA^{Y}	gagggcctatttcccatgattccttcatatttgcatatacgatacaaggctgttagagagataattggaattaatttgactgtaaacacaaagatattagtacaaaatacgtga<br>cgtagaaagtaataatttcttgggtagtttgcagttttaaaattatgttttaaaatggactatcatatgcttaccgtaacttgaaagtatttcgatttcttggctttatatatcttgtgg<br>aaaggacgaaacaccgCCTTCGATAGCTCAGTTGGTAGAGCGGAGGACTTTAGATCCTTAGGTCGCTGGTTCG<br>AATCCGGCTCGAAGGAtttttttaaagaattgagactagcctcgagcggccgccccccttcaccgagggcctatttcccatgattccttcatatttgcatatac<br>gatacaaggctgttagagagataattggaattaatttgactgtaaacacaaagatattagtacaaaatacgtgacgtagaaagtaataatttcttgggtagtttgcagttttaa<br>aattatgttttaaaatggactatcatatgcttaccgtaacttgaaagtatttcgatttcttggctttatatatcttgtggaaaggacgaaacaccgCCTTCGATAGCT<br>CAGTTGGTAGAGCGGAGGACTTTAGATCCTTAGGTCGCTGGTTCGAATCCGGCTCGAAGGAtttttttaaaga<br>attctcgacctcgagacaaatggcagtattcatccacaattttaaaagaaaaggggggattggggggtacagtgcaggggaaagaatagtagacataatagcaacaga<br>catacaaactaaagaattacaaaaacaaattacaaaaattcaaaattttcgggtttattacagggacagcagagatccactttggccgcggctcgagggggttggggttg<br>cgccttttccaaggcagccctgggtttgcgcagggacgcggctgctctgggcgtggttccgggaaacgcagcggcgccgaccctgggactcgcacattcttcacgtc<br>cgttcgcagcgtcacccggatcttcgccgctacccttgtgggccccccggcgacgcttcctgctccgcccctaagtcgggaaggttccttgcggttcgcggcgtgccg<br>gacgtgacaaacggaagccgcacgtctcactagtaccctcgcagacggacagcgccagggagcaatggcagcgcgccgaccgcgatgggctgtggccaatagcg<br>gctgctcagcagggcgcgccgagagcagcggccgggaaggggcggtgcgggaggcggggtgtggggcggtagtgtgggccctgttcctgcccgcgcggtgttc<br>cgcattctgcaagcctccggagcgcacgtcggcagtcggctccctcgttgaccgaatcaccgacctctctccccaggggggatccatggtgagcaagggcgaggagc<br>tgttcaccggggtggtgcccatcctggtcgagctggacggcgacgtaaacggccacaagttcagcgtgtccggcgagggcgagggcgatgccacctacggcaagc<br>tgaccctgaagttcatctgcaccaccggcaagctgcccgtgccctggcccaccctcgtgaccaccctgacctacggcgtgcagtgcttcagccgctaccccgaccaca<br>tgaagcagcacgacttcttcaagtccgccatgcccgaaggctacgtccaggagcgcaccatcttcttcaaggacgacggcaactacaagacccgcgccgaggtgaa<br>gttcgagggcgacaccctggtgaaccgcatcgagctgaagggcatcgacttcaaggaggacggcaacatcctggggcacaagctggagtacaactacaacagcca<br>caacgtctatatcatggccgacaagcagaagaacggcatcaaggtgaacttcaagatccgccacaacatcgaggacggcagcgtgcagctcgccgaccactaccag<br>cagaacacccccatcggcgacggccccgtgctgctgcccgacaaccactacctgagcacccagtccgccctgagcaaagaccccaacgagaagcgcgatcacatg<br>gtcctgctggagttcgtgaccgccgccgggatcactctcggcatggacgagctgtacaagtaa

Table S3. Statistical analysis of full-length dystrophin expression levels across different treatment groups in various muscle tissues.

Muscle Type	Comparison Groups	Mean Difference (%)	95% CI [Lower, Upper]	Adjusted p-value	Significance
Heart	WT vs mdx+saline	99.94	[91.91,108.0]	<0.0001	****
Heart	WT vs mdx+sup-tRNA^{Y}	53.46	[45.43,61.50]	<0.0001	****
Heart	WT vs mdx+sup-tRNA^{Y}+CC-90009	38.57	[30.53,46.60]	<0.0001	****
Heart	WT vs mdx+CC-90009	99.85	[91.82,107.9]	<0.0001	****
Heart	mdx+saline vs mdx+sup-tRNA^{Y}	-46.48	[-54.51, -38.44]	<0.0001	****
Heart	mdx+saline vs mdx+sup-tRNA^{Y}+CC-90009	-61.37	[-69.41, -53.34]	<0.0001	****
Heart	mdx+saline vs mdx+CC-90009	-0.08869	[-8.123,7.946]	>0.9999	ns
Heart	mdx+sup-tRNA^{Y} vs mdx+sup-tRNA^{Y}+CC-90009	-14.89	[-22.93, -6.859]	<0.0001	****
Heart	mdx+sup-tRNA^{Y} vs mdx+CC-90009	46.39	[38.36,54.42]	<0.0001	****
Heart	mdx+sup-tRNA^{Y}+CC-90009 vs mdx+CC-90009	61.28	[53.25,69.32]	<0.0001	****
Gastrocnemius	WT vs mdx+saline	99.94	[91.91,108.0]	<0.0001	****
Gastrocnemius	WT vs mdx+sup-tRNA^{Y}	88.38	[80.35,96.42]	<0.0001	****
Gastrocnemius	WT vs mdx+sup-tRNA^{Y}+CC-90009	82.04	[74.00,90.07]	<0.0001	****
Gastrocnemius	WT vs mdx+CC-90009	99.85	[91.82,107.9]	<0.0001	****

Gastrocnemius	mdx+saline vs mdx +mdx+sup-tRNA^{Y}	-11.56	[-19.59, -3.523]	0.0017	**
Gastrocnemius	mdx+saline vs mdx +sup-tRNA^{Y}+CC-90009	-17.9	[-25.94, -9.867]	<0.0001	****
Gastrocnemius	mdx+saline vs mdx +CC-90009	-0.08869	[-8.123,7.946]	>0.9999	ns
Gastrocnemius	mdx+sup-tRNA^{Y} vs mdx+sup-tRNA^{Y}+ CC-90009	-6.345	[-14.38,1.690]	0.1809	ns
Gastrocnemius	mdx +sup-tRNA^{Y} vs mdx+CC-90009	11.47	[3.434,19.50]	0.0019	**
Gastrocnemius	mdx+sup-tRNA^{Y}+CC-90009 vs mdx+CC-90009	17.81	[9.779,25.85]	<0.0001	****
Quadriceps	WT vs mdx+saline	99.94	[91.91,108.0]	<0.0001	****
Quadriceps	WT vs mdx+mdx+ sup-tRNA^{Y}	88.79	[80.76,96.83]	<0.0001	****
Quadriceps	WT vs mdx+sup-tRNA^{Y}+CC-90009	91.52	[83.49,99.56]	<0.0001	****
Quadriceps	WT vs mdx+CC-90009	99.85	[91.82,107.9]	<0.0001	****
Quadriceps	mdx+saline vs mdx +sup-tRNA^{Y}	-11.15	[-19.18, -3.114]	0.0026	**
Quadriceps	mdx+saline vs mdx +sup-tRNA^{Y}+CC-90009	-8.417	[-16.45,-0.3825]	0.036	*
Quadriceps	mdx+saline vs mdx +CC-90009	-0.08869	[-8.123,7.946]	>0.9999	ns
Quadriceps	mdx +sup-tRNAY vs mdx+sup-tRNA^{Y} +CC-90009	2.731	[-5.303,10.77]	0.8666	ns
Quadriceps	mdx +sup-tRNA^{Y} vs mdx+CC-90009	11.06	[3.025,19.09]	0.0029	**

Quadriceps	mdx+sup-tRNA^{Y}+CC-90009 vs mdx+CC-90009	8.328	[0.2938,16.36]	0.0389	*
Triceps	WT vs mdx+saline	99.94	[91.91,108.0]	<0.0001	****
Triceps	WT vs mdx+sup-tRNA^{Y}	95.13	[87.09,103.2]	<0.0001	****
Triceps	WT vs mdx+sup-tRNA^{Y}+CC-90009	94.28	[86.25,102.3]	<0.0001	****
Triceps	WT vs mdx+CC-90009	99.85	[91.82,107.9]	<0.0001	****
Triceps	mdx+saline vs mdx+sup-tRNA^{Y}	-4.815	[-12.85,3.220]	0.4387	ns
Triceps	mdx+saline vs mdx+sup-tRNA^{Y}+CC-90009	-5.66	[-13.69,2.374]	0.2789	ns
Triceps	mdx+saline vs mdx+CC-90009	-0.08869	[-8.123,7.946]	>0.9999	ns
Triceps	mdx+sup-tRNA^{Y} vs mdx+sup-tRNA^{Y}+CC-90009	-0.8457	[-8.880,7.189]	0.9981	ns
Triceps	mdx+sup-tRNA^{Y} vs mdx+CC-90009	4.726	[-3.309,12.76]	0.4574	ns
Triceps	mdx+sup-tRNA^{Y}+CC-90009 vs mdx+CC-90009	5.571	[-2.463,13.61]	0.2938	ns

One-way ANOVA was first performed to evaluate differences in full-length dystrophin expression among treatment groups in each muscle type, and significant overall differences were observed ($P < 0.0001$). Subsequently, Tukey's multiple comparisons test was conducted to determine pairwise differences between specific groups. This table summarizes the mean differences, 95% confidence intervals, adjusted p-values, and significance levels.

Significance levels: ns = not significant; $P < 0.05$ (*), $P < 0.01$ (**), $P < 0.001$ (***), $P < 0.0001$ (****).

Referencce：

1. Roy B, Friesen WJ, Tomizawa Y, et al. Ataluren stimulates ribosomal selection of near-cognate tRNAs to promote nonsense suppression. *Proc Natl Acad Sci U S A.* 2016;113(44):12508-12513.
2. Ng MY, Li H, Ghelfi MD, Goldman YE, Cooperman BS. Ataluren and aminoglycosides stimulate read-through of nonsense codons by orthogonal mechanisms. *Proc Natl Acad Sci U S A.* 2021;118(2).
3. Huang S, Bhattacharya A, Ghelfi MD, et al. Ataluren binds to multiple protein synthesis apparatus sites and competitively inhibits release factor-dependent termination. *Nat Commun.* 2022;13(1):2413.
4. Surka C, Jin L, Mbong N, et al. CC-90009, a novel cereblon E3 ligase modulator, targets acute myeloid leukemia blasts and leukemia stem cells. *Blood.* 2021;137(5):661-677.
5. Baradaran-Heravi A, Balgi AD, Hosseini-Farahabadi S, Choi K, Has C, Roberge M. Effect of small molecule eRF3 degraders on premature termination codon readthrough. *Nucleic Acids Res.* 2021;49(7):3692-3708.
6. Sharma J, Du M, Wong E, et al. A small molecule that induces translational readthrough of CFTR nonsense mutations by eRF1 depletion. *Nature Communications.* 2021;12(1).
7. Coelho JPL, Yip MCJ, Oltion K, Taunton J, Shao S. The eRF1 degrader SRI-41315 acts as a molecular glue at the ribosomal decoding center. *Nat Chem Biol.* 2024;20(7):877-884.
8. van der Wal E, Herrero-Hernandez P, Wan R, et al. Large-Scale Expansion of Human iPSC-Derived Skeletal Muscle Cells for Disease Modeling and Cell-Based Therapeutic Strategies. *Stem Cell Reports.* 2018;10(6):1975-1990.
9. Lian X, Bao X, Zilberter M, et al. Chemically defined, albumin-free human cardiomyocyte generation. *Nature Methods.* 2015;12(7):595-596.
10. Chal J, Oginuma M, Al Tanoury Z, et al. Differentiation of pluripotent stem cells to muscle fiber to model Duchenne muscular dystrophy. *Nat Biotechnol.* 2015;33(9):962-969.
11. Arechavala-Gomeza V, Kinali M, Feng L, et al. Immunohistological intensity measurements as a tool to assess sarcolemma-associated protein expression. *Neuropathol Appl Neurobiol.* 2010;36(4):265-274.
12. Wu R, Li P, Xiao P, et al. Activation of endogenous full-length utrophin by MyoAAV-UA as a therapeutic approach for Duchenne muscular dystrophy. *Nat Commun.* 2025;16(1):2398.
13. Wang J, Zhang Y, Mendonca CA, et al. AAV-delivered suppressor tRNA overcomes a nonsense mutation in mice. *Nature.* 2022;604(7905):343-348.
14. Lueck JD, Yoon JS, Perales-Puchalt A, et al. Engineered transfer RNAs for suppression of premature termination codons. *Nat Commun.* 2019;10(1):822.
